# Heart Disease and Thoracic Aneurysm

**Published:** 1908-09

**Authors:** 


### Reviews of Books.

### BOOKS ON MEDICINE.

**Heart Disease and Thoracic Aneurysm.** By F. J. POYNTON, M.D. Pp. x., 310. London: Oxford Medical Publications. 1907.

It is difficult nowadays to write a short book on diseases of the heart, and at the same time to deal adequately with the subject. Dr. Poynton, in this manual, has attained a certain measure of success in his task, but it seems to us that he has tried to give too much detail without sufficient explanation. The section on rheumatic heart disease is, as would be expected, the best. Dr. Poynton describes this form of heart disease as due to infection by the diplococcus rheumaticus, but justly observes that, if this etiology be not accepted, the facts that are stated about the morbid anatomy still hold good. The chapters on chronic valvular disease and on myocardial affections are also good, and likely to prove helpful to the student. The advice as to treatment is in all cases sound. We think, however, that the dose given for erythrol tetranitrate as an initial one is too large. Some patients are very susceptible to this drug, and made uncomfortable by it unless the dose is a small one. The author does not believe that the salicyl compounds have a specific action against the rheumatic poison. The subjects of arrhythmia and irregularities of the cardiac action, and the methods of studying them by polygraphic tracings, receive very inadequate treatment, which is somewhat surprising in a new book on heart disease, considering the large amount of work that has recently been done upon them, and their importance from the light they throw upon the heart's action in disease. We think also that the student should be recommended to learn the use of the straight stethoscope, and that the beginner will find the heart sounds both more distinct and more easily timed with it than with the binaural. The section on aneurysm is very short. We can only suppose that this is intentional, and that the author thought it best to give only a short sketch of the most important features. We think that the duration of a rupture of an intrapericardial aneurysm into the pulmonary artery is to be measured by weeks rather than months. Attacks of so-called "asthma" should also be mentioned as often an early symptom of aneurysm in this situation.

The book is clearly written, but suffers from over-condensation. The chief features of the different forms of heart disease are well brought out, and the advice as to treatment, as stated above, is uniformly sound. No doubt the student who masters this book will have a good basis to go upon for his clinical work. Appendices contain prescriptions, diets, and directions for Schott exercises and Nauheim Baths.

